# Aerobic Exercise Training Prevents Perivascular Adipose Tissue-Induced Endothelial Dysfunction in Thoracic Aorta of Obese Mice

**DOI:** 10.3389/fphys.2019.01009

**Published:** 2019-08-16

**Authors:** Andressa S. Sousa, Amanda C. S. Sponton, César B. Trifone, Maria A. Delbin

**Affiliations:** Laboratory of Vascular Biology, Department of Structural and Functional Biology, Institute of Biology, University of Campinas (UNICAMP), Campinas, Brazil

**Keywords:** obese mice, perivascular adipose tissue, endothelial dysfunction, aerobic exercise training, vascular reactivity

## Abstract

**Background:** The mechanisms underlying the perivascular adipose tissue (PVAT) dysfunction in obesity are closely related to inflammation and oxidative stress. The present study aimed to investigate the effects of aerobic exercise training on PVAT-induced endothelial dysfunction of thoracic aorta of obese mice.

**Methods:** Male mice C57BL6/JUnib (6–7 weeks) were divided into: sedentary (c-SD), trained (c-TR), obese sedentary (o-SD), and obese trained (o-TR). Obesity was induced by 16 weeks of high-fat diet and exercise training of moderate intensity started after 8 weeks of protocol and was performed on a treadmill, 5 days/week, for more 8 weeks, 60 min per session. The vascular responsiveness was performed in thoracic aorta in the absence (PVAT−) or in the presence (PVAT+) of PVAT. We analyzed circulatory parameters, protein expression, vascular nitric oxide (NO) production, and reactive oxygen species (ROS) in PVAT.

**Results:** The maximal responses to acetylcholine (ACh) were reduced in PVAT+ compared with PVAT− rings in the o-SD group, accompanied by an increase in circulating glucose, insulin, resistin, leptin, and TNF-α. Additionally, the protein expression of iNOS and generation of ROS were increased in PVAT and production of vascular NO was reduced in the o-SD group compared with c-SD. In the o-TR group, the relaxation response to ACh was completely restored and the circulatory TNF-α, iNOS protein expression, and ROS were normalized with increased expression of Mn-SOD in PVAT, resulting in enhanced vascular NO production.

**Conclusion:** The PVAT-induced endothelial dysfunction in thoracic aorta of obese mice, associated with circulatory inflammation and oxidative stress. Aerobic exercise training upregulated the anti-oxidant expression and decreased PVAT oxidative stress with beneficial impact on endothelium-dependent relaxation.

## Introduction

The unique and specialized paracrine/endocrine function of perivascular adipose tissue (PVAT) to regulate vascular responsiveness has been recently identified. The first studies demonstrated an anti-contractile effect of PVAT on the vascular response to norepinephrine (Soltis and Cassis, 1991), angiotensin II, serotonin, and phenylephrine in lean animals without any alteration in the relaxation response to acetylcholine and sodium nitroprusside (Soltis and Cassis, 1991; Lohn et al., 2002; Gollasch and Dubrovska, 2004). Further studies in obese animals showed a loss of the PVAT anti-contractile effect (Ma et al., 2010; Payne et al., 2010) and in contrast, it was demonstrated an adaptive alteration of PVAT in obese animals with enhanced anti-contractile response aimed to preserve the endothelial function (Gil-Ortega et al., 2010; Araujo et al., 2018). Lastly, other studies unveiled an association of endothelial dysfunction and PVAT in obese animals (Ketonen et al., 2010; Xia and Li, 2017; DeVallance et al., 2018), showing a potential adipose dysfunction of PVAT and a complex mechanism involving inflammation, increased production of reactive oxygen species (ROS), and alterations in endothelial nitric oxide synthase (eNOS) pathways (Bailey-Downs et al., 2013; Xia et al., 2016).

Obesity has diverse adverse effects on cardiovascular system (Montero et al., 2012). In this condition, the adipose tissue becomes dysfunctional and shifts toward a pro-inflammatory and pro-oxidative state linked to endothelial dysfunction (de Mello et al., 2018). Studies have suggested that the hypoxia, inflammation, and oxidative stress of PVAT can be the fundamental mechanism involved in PVAT dysfunction in obesity (Chatterjee et al., 2009; Greenstein et al., 2009; Gil-Ortega et al., 2014; Xia and Li, 2017). The high amount of PVAT in obesity is accompanied by macrophage infiltration, increased monocyte chemoattractant protein-1 (MCP-1), and reduced adiponectin secretion (Bailey-Downs et al., 2013). Thus, the pro-inflammatory cytokines generated in the PVAT stimulates the oxidative stress and ROS generation to endothelial cell and decreases nitric oxide (NO) bioavailability and the relaxation response (Tesauro et al., 2008; Virdis, 2016).

Evidence has shown that physical inactivity is the fourth leading risk factor cause of global deaths and the major risk factor for the development of overweight and obesity (World Health Organization, 2010; Bull et al., 2017). Aerobic exercise training, continuously performed, promotes beneficial effects regarding prevention and treatment of cardiovascular and metabolic diseases (Buttar et al., 2005; Agarwal, 2012). The activation of mechanosensors of endothelial cells, during exercise, directly stimulates eNOS and NO production (Gielen et al., 2010). It was also demonstrated, in different studies and protocols of aerobic exercise training, that the reduction in vascular oxidative stress and increased anti-oxidant response result in enhanced NO bioavailability (Delbin et al., 2012; Sponton et al., 2017). In addition, the beneficial effects of aerobic exercise were related to the ability to alter metabolic phenotype and inflammatory biomarkers of adipose tissue (Kawanishi et al., 2010; De Matteis et al., 2013; Boa et al., 2017). Recent studies demonstrated beneficial effects of exercise training to restore eNOS activation (Meziat et al., 2019) or reducing iNOS protein expression in PVAT (Araujo et al., 2018) both linked to normalization of contractile vascular reactivity in obese rats.

The purpose of the study is to investigate the effects of aerobic exercise training on PVAT-induced endothelial dysfunction of the thoracic aorta of obese mice. The hypothesis of the study is that aerobic exercise training causes modifications on the PVAT oxidative stress (increases the antioxidant response and decreases ROS generations) positively affecting NO production and bioavailability and the endothelium-dependent relaxation response.

## Materials and Methods

### Animal Model

The study was approved by the Ethics Committee for Animal Use (CEUA 3278-1) at the University of Campinas (UNICAMP) stated by the Brazilian Society of Laboratory Animal Science. Male C56BL/6/JUnib mice (weighing 20–24 g) from the Animal Care Facility of UNICAMP were maintained in a room at 20–21°C with a normal light/dark cycle. Food and water were provided ad libitum to all animals. The animals were housed in two or three animals per cage and were separated into the experimental groups: sedentary (c-SD), trained (c-TR), obese sedentary (o-SD), and obese trained (o-TR). For 16 weeks, animals in the c-SD and c-TR groups were fed standard chow (3.6 kcal/g), whereas animals in the o-SD and o-TR groups were fed a high-fat diet (6.2 kcal/g: 32% carbohydrates, 20.3% proteins, and 38% lipids, PragSoluções Biociências, Brazil). The animals in the c-TR and o-TR groups, initiated aerobic exercise training after 8 weeks of study, which was maintained until the end of the protocol (for more 8 weeks). The food intake measurement was performed weekly during all the study.

### Exercise Program

Animals were trained on a treadmill designed for small animals (Gesan, Brazil). The exercise program consisted of sessions of 60 min/day, 5 days/week, at a 0% grade and at 50–60% of the maximal speed, determined using an incremental protocol previously described (Sponton et al., 2017), for 8 weeks. In the first week of the training program, the duration and speed started at 10 m/min for 30 min and were progressively increased to 15 m/min for 60 min. From the second to eighth week, the training program consisted of 10 min at 40% of maximal speed, 40 min at 50–60% of maximal speed, and 10 min at 40% of maximal speed (total of 60 min/day). All animals were trained between 6:00 and 8:00 a.m.

All groups of animals were submitted again to acute incremental exercise testing on the treadmill during the last week of the study to evaluate the effectiveness of the training program.

### Biochemical Profile

At 48 h after the last exercise training session and after 12 h of fasting, blood glucose was measured from the tail vein using standard test strips (Accu-Chek Advantage, Roche Diagnostics, USA). Immediately after, the animals were anesthetized (urethane 2 g/kg, i.p.), and blood samples were collected from the cardiac puncture and centrifuged (8,000 *g* for 15 min). Total cholesterol (TC) levels were measured in fresh serum samples using standard commercial kits (Roche Diagnostic GmbH, Germany). Serum levels of insulin, leptin, resistin, tumor necrosis factor-alpha (TNF-α), and total adiponectin were determined using a commercially available ELISA kit (Millipore Corporation, USA, catalogue number: EZHI-14 K; R&D Systems, USA, catalogue number: DLP00, DRSN00, DTA00C and DRP300, respectively).

### Tissue Collection and Vascular Function

The animals were euthanized and the epididymal fat pad was collected and weighed. Isolated aortic rings procedure was performed as previously described (Araujo et al., 2018). Briefly, thoracic aorta was cut into rings of 2 mm in the absence (PVAT−) or in the presence (PVAT+) of PVAT. Each ring was mounted in a myograph chamber (model 610 M; Danish Myo Technology, Denmark) under a resting tension of 5 millinewtons (mN), as previously described (Sponton et al., 2017).

The vascular viability and maximal contraction were determined by replacing Krebs to KCl 80 mM. Next, the rings were deeply washed with Krebs and cumulative relaxation-response curves to acetylcholine (ACh, 1 nM–100 μM) and sodium nitroprusside (SNP, 100 pM–100 μM) were obtained after precontraction with a thromboxane A2 analog (U46619, at a concentration necessary to produce 50–80% of the maximal contraction of 80 mM KCl). Relaxation responses were plotted as a percentage of the contraction induced by U46619. Contractile concentration-response curves were also obtained to U46619 (1 nM–10 μM). Contractile responses were plotted according to the force and length from each ring as millinewton per millimeter (mN/mm). After vascular responsiveness, PVAT was collected, the excess of Krebs solution was dried with filter paper and the tissue was weighted wet and measured as milligram/millimeter (mg/mm).

All concentration-response data were evaluated for a fit to a logistics function, according to previous study (Van Rossum, 1963). The responses for each agonist are shown as the mean ± SEM of maximum response (*E*_MAX_) and potency (pEC_50_).

### Western Blotting

The expression of proteins was determined by Western blotting in thoracic aorta and in the respective PVAT tissue lysates using techniques previously described (Delbin et al., 2012). Briefly, proteins from homogenized aorta (50 μg) and PVAT (100 μg) were separated by polyacrylamide-SDS gels and transferred onto PVDF membranes. Membranes were incubated overnight at 4°C with primary antibodies: mouse monoclonal anti-endothelial NO synthase (eNOS 1:1000, BD Transduction, USA), mouse monoclonal anti-inducible NO synthase (iNOS 1:1000, BD Transduction, USA, only in PVAT), anti-Cu/Zn superoxide dismutase (Cu/Zn-SOD 1:1000, Sigma-Aldrich), anti-extracellular superoxide dismutase (Ec-SOD 1:1000, Enzo Life Sciences, USA), and anti-manganese superoxide dismutase (Mn-SOD 1:1000, Axxora LLC, USA). The chemiluminescent detection was carried out using ECL (Thermo Scientific, USA) and Image Quant LAS 4000 (GE Healthcare, USA) hardware and software. The intensity of the bands was quantified using ImageJ 1.46p software (National Institutes of Health, USA). The membranes were used to determine α-actin (for aorta) or α-tubulin (for PVAT) protein expression as an internal control using a mouse monoclonal anti-α-actin (1:5000 Abcam, USA) and anti-α-tubulin (1:1000 Santa Cruz Biotechnology Inc., USA). The results from all groups are presented relative to those of the c-SD group (c-SD = 1).

### Vascular Nitric Oxide

The thoracic aorta PVAT+ segments were prepared as previously described (Delbin et al., 2012) to measure NO production using the NO-sensitive fluorescent dye 4,5-diaminofluorescein diacetate (DAF-2 8 μM, Sigma-Aldrich). The tissue sample were stimulated without (basal) or with ACh (30 μM). Images were obtained with an optical microscope (Eclipse 80i, Nikon, Japan) equipped with fluorescein filter and camera (DS-U3, Nikon, Japan), using a 10X objective. The images were analyzed using the Image J 1.46p software (National Institute of Health). The results are expressed as the difference of ACh stimulation integrative density and basal integrative density. The results from all groups are presented relative to those of the c-SD group (c-SD = 1).

The limitation of DAF-2 is related to uncertain specify for being be enzymatically converted into variety of highly fluorescent derivatives. DAF react not only with NO but with intermediate formed in the course of the oxygen oxidation of NO to NO_2_^−^. In addition, the reaction speed of NO oxidation is slow and the chemical yield of the product is low; thus, the rate at which NO is oxidized imposes an intrinsic limitation on detection by this method (Kojima et al., 1998).

### Reactive Oxygen Species

Thoracic aorta PVAT+ segments were prepared as previously described (Davel et al., 2012) to evaluate in ROS generation using the oxidative fluorescent dye hydroethidine (DHE, Invitrogen, USA). Images were obtained with an optical microscope equipped with filter to rhodamine and camera (DS-U3, Nikon), using a 10X objective. The number of ethidium bromide-positive nuclei was automatically counted in thoracic aorta and in the respective PVAT using Image J software (National Institutes of Health) and expressed as labeled per square millimeter (nuclei/mm^2^). The results from all groups are presented relative to those of the c-SD group (c-SD = 1).

Limitations of DHE are related to the semi-quantitative assay, since only a fraction of O_2_^−^ or H_2_O_2_ is detected (Kalyanaraman et al., 2017). Moreover, the major drawback of DHE related probes is poor selectivity toward O_2_^−^ and the experiment procedures must be performed in dim light because the probes are light sensitive (Wong, 2016).

### Histology

Thoracic PVAT transverse sections (5 μm) were obtained as previously described (Araujo et al., 2018). The images were obtained with inverted microscope equipped with digital camera and NIS Elements Basic Research (3.2 Software, Nikon, Japan), using a 40X objective.

### Statistical Analyses

Data are expressed as mean ± SEM. For comparison of experimental groups, two-way ANOVA followed by Bonferroni’s *post hoc* and the Student’s *t*-test were used (Instat Software, GraphPad Prism, USA). A value of *p* < 0.05 was considered statistically significant.

### Chemicals

Acetylcholine chloride, sodium nitroprusside dihydrate, and thromboxane A2 analog were purchased from Sigma-Aldrich Co. (Saint Louis, USA).

## Results

All groups presented similar responses to the incremental exercise test before the exercise training (Table 1). After the exercise training, we verified an improvement in physical performance as assessed by total time (min), total distance (meters), and maximal speed (meters/min) in the c-TR and o-TR group when compared with the c-SD and o-SD groups, respectively. When we compared only the trained groups, we observed a decreased performance in o-TR group compared with c-TR group (Table 1).

**Table 1:** Incremental exercise test performed before the exercise training (BEFORE) and at the end (FINAL) of the exercise training from sedentary (c-SD), trained (c-TR), obese sedentary (o-SD), and obese trained (o-TR) mice.

	Before				Final			
	c-SD (14)	c-TR (15)	o-SD (16)	o-TR (15)	c-SD (14)	c-TR (15)	o-SD (16)	o-TR (15)
Time (min)	19.4 ± 0.6	17.8 ± 0.7	18.9 ± 0.8	17.7 ± 0.5	15.6 ± 1.3	31.8 ± 1.1^*^	15.7 ± 0.8	25.3 ± 0.2^+#^
Distance (meters)	276 ± 16	235 ± 18	267 ± 21	236 ± 13	220 ± 33	825 ± 53^*^	219 ± 21	526 ± 9^+#^
Max Speed (m/min)	24.6 ± 1.0	26.4 ± 1.0	24.6 ± 1.0	24.6 ± 1.0	19.8 ± 2.0	38.4 ± 2.0^*^	20.4 ± 1.0	31.2 ± 1.0^+#^

Data are mean ± SEM. The number of animals per group is indicated in the parentheses. Two-way ANOVA: ^*^p < 0.05 compared with the c-SD; ^#^p < 0.05 compared with the c-TR; ^+^p < 0.05 compared with the o-SD.

Initial body weights were similar in all groups (Table 2). The o-SD group presented a significant increase in food intake (31%), body weight (70%), epididymal fat pad (370%), PVAT (320%), total cholesterol (240%), blood glucose (65%), and insulin (740%) levels, when compared with the c-SD group (Table 2). Exercise training was effective to decrease only the insulin levels in the o-TR group when compared with the o-SD group.

**Table 2:** Body weight, epididymal fat pad, perivascular adipose tissue (PVAT), food intake, and metabolic biomarkers in mice from sedentary (c-SD), trained (c-TR), obese sedentary (o-SD), and obese trained (o-TR).

	c-SD	c-TR	o-SD	o-TR
I-Body weight (g)	20.8 ± 0.8 (14)	20.7 ± 0.8 (15)	20.7 ± 0.6 (16)	20.3 ± 0.7 (15)
F-Body weight (g)	27.4 ± 1.0 (14)	27.5 ± 0.8 (15)	46 ± 1.1^*^ (16)	45 ± 1.2^#^ (15)
Epididymal fat (g)	0.3 ± 0.03 (14)	0.3 ± 0.01 (15)	1.4 ± 0.09^*^ (16)	1.5 ± 0.09^#^ (15)
Food intake (kcal/animal/day)	14.2 ± 0.4 (14)	12.9 ± 0.4 (15)	18.6 ± 0.4^*^ (16)	17.0 ± 0.4^#^ (15)
PVAT (mg/mm)	0.8 ± 0.2 (14)	0.7 ± 0.2 (15)	3.0 ± 0.5^*^ (16)	2.7 ± 0.1^#^ (15)
Total cholesterol (mg/dl)	82 ± 4.5 (7)	85.6 ± 2 (6)	278 ± 45^*^ (10)	212 ± 8^#^ (10)
Blood glucose (mg/dl)	95 ± 6 (14)	106 ± 7.2 (15)	155 ± 10^*^ (16)	148 ± 7.8^#^ (15)
Insulin (ng/ml)	1.0 ± 0.3 (8)	1.5 ± 0.3 (6)	8.3 ± 0.9^*^ (10)	3.9 ± 1.3^+^ (7)

Initial (I); Final (F). Data are mean ± SEM. The number of animals per group is indicated in the parentheses. Two-way ANOVA: ^*^p < 0.05 compared with the c-SD; ^#^p < 0.05 compared with the c-TR; ^+^p < 0.05 compared with the o-SD.

**Figure 1:**
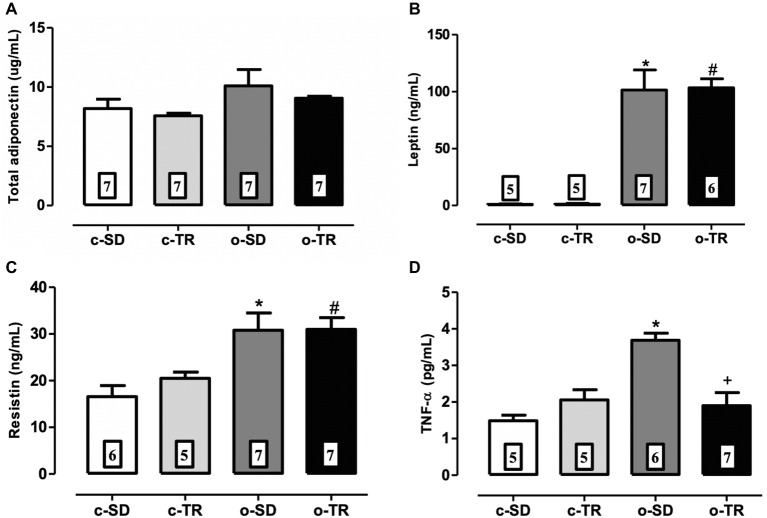
Serum levels of total adiponectin **(A)**, leptin **(B)**, resistin **(C),** and TNF-α **(D)** in mice from sedentary (c-SD), trained (c-TR), obese sedentary (o-SD), and obese trained (o-TR) groups. Data are presented as mean ± SEM. The number of animals per group is indicated in the figure. Two-way ANOVA: ^*^p < 0.05 compared with the c-SD; ^#^*p* < 0.05 compared with the c-TR; ^+^*p* < 0.05 compared with the o-SD.

No changes were observed in any group regarding levels of adiponectin (Figure 1A). The levels of pro-inflammatory cytokines leptin, resistin, and TNF-α were markedly increased in the o-SD group (Figures 1B–D) when compared with c-SD group. Exercise training restored the TNF-α levels in the o-TR group (Figure 1D).

The maximal contractile responses to KCl 80 mmol/L (mN/mm) did not change in all groups, independent of PVAT (c-SD PVAT−: 12.0 ± 1.0 vs. PVAT+: 11.0 ± 1.0, *n* = 14; c-TR PVAT−: 11.0 ± 1.0 vs. PVAT+: 11.0 ± 1.0, *n* = 15; o-SD PVAT−: 12.0 ± 1.0 vs. PVAT+: 10.0 ± 1.0, *n* = 16; o-TR PVAT−: 10.0 ± 1.0 vs. PVAT+: 9.0 ± 1.0, *n* = 15).

**Figure 2:**
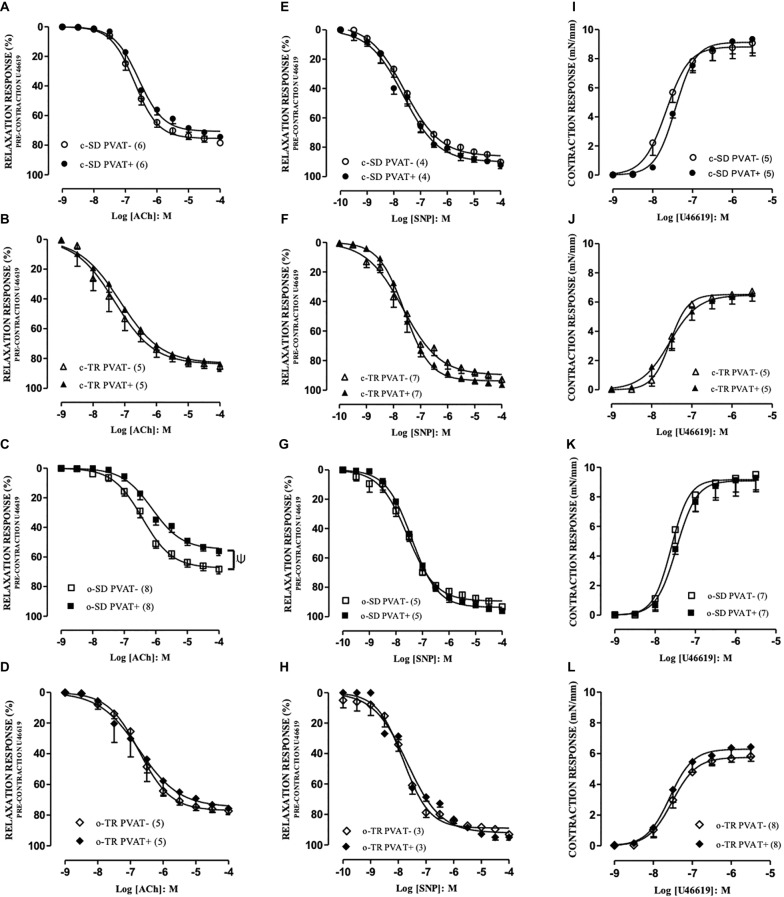
Concentration-response curves to acetylcholine (ACh: **A–D**), sodium nitroprusside (SNP: **E–H**) and thromboxane A2 analog (U46619: **I–L**) in aortic rings. Groups: sedentary (c-SD PVAT−, c-SD PVAT+), trained (c-TR PVAT−, c-TR PVAT+), obese sedentary (o-SD PVAT− and o-SD PVAT+), and obese trained (o-TR PVAT− and PVAT+). Data are presented as mean ± SEM. The number of animals per group is indicated in the figure. Unpaired Student’s *t*-test: ^Ψ^*p* < 0.05 *E*_MAX_ PVAT+ vs. respective *E*_MAX_ PVAT–.

The agents ACh and SNP produced relaxation responses in PVAT– and PVAT+ aortic rings with intact endothelium. In c-SD and c-TR groups no alteration was verified for maximal responses (*E*_MAX_, Figures 2A,B) for ACh in PVAT+ compared with PVAT− rings. On the other hand, the *E*_MAX_ values for ACh were decreased in o-SD PVAT+ rings (12%) when compared with the respective PVAT− rings (Figure 2C), whereas aerobic exercise training restored the reduction in the *E*_MAX_ in o-TR PVAT+ compared with the respective PVAT– rings (Figure 2D).

When we compared only the PVAT− rings, the *E*_MAX_ (10%) values were decreased in o-SD group when compared with c-SD group, and these alterations were enhanced in PVAT+ rings (19%) (Table 3). Exercise training (o-TR) completely restored the reduction in the *E*_MAX_ when compared with o-SD (Table 3). The exercise training increased the EC_50_ in c-TR group in rings PVAT− (4.3-fold) when compared with c-SD group without alterations in *E*_MAX_ (Table 3). When we compared only the trained groups the EC_50_ was decreased in rings PVAT− (5.5 fold) in o-TR when we compared with c-TR group, *E*_MAX_ values were not affected (Table 3). No alteration was observed in *E*_MAX_ (Figures 2E–H) and pEC_50_ values (data not shown) in any group for SNP.

**Table 3:** Maximal response (*E*_MAX_) and potency values (pEC_50_) obtained from concentration-response curves to acetylcholine (ACh) and U44619 in mice thoracic aorta PVAT− and PVAT+ from sedentary (c-SD), trained (c-TR), obese sedentary (o-SD), and obese trained (o-TR) groups.

		c-SD	c-TR	o-SD	o-TR
ACh	*E*_MAX_ (%)	78.4 ± 1.0 (6)	86 ± 3 (5)	68 ± 3^*^ (8)	78 ± 3^+^ (5)
PVAT-	pEC_50_	6.71 ± 0.08	7.35 ± 0.17^*^	6.46 ± 0.08	6.61 ± 0.04^#^
ACh	*E*_MAX_ (%)	75 ± 1.5 (7)	84 ± 3.8 (5)	56 ± 2.9^*^ (8)	75 ± 3.8^**+**^ (5)
PVAT+	pEC_50_	6.60 ± 0.10	7.21 ± 0.15	6.07 ± 0.14	6.83 ± 0.37
U46619	*E*_MAX_ (mN/mm)	9.1 ± 0.9 (5)	6.7 ± 0.3^*^ (5)	9.5 ± 1.1 (7)	5.8 ± 0.3^**+**^ (8)
PVAT-	pEC_50_	7.7 ± 0.09	7.5 ± 0.09	7.6 ± 0.05	7.6 ± 0.11
U46619	*E*_MAX_ (mN/mm)	9.3 ± 0.9 (5)	6.9 ± 0.5^*^ (5)	9.3 ± 0.8 (7)	6.4 ± 0.6^**+**^ (8)
PVAT+	pEC_50_	7.4 ± 0.06	7.5 ± 0.15	7.4 ± 0.08	7.6 ± 0.09

Potency is represented as −log of molar concentration to produce 50% of the maximal responses. Data are mean ± SEM. The number of animals per group is indicated in the parentheses. Two-way ANOVA: ^*^p < 0.05 compared with the c-SD; ^**+**^p < 0.05 compared with the o-SD; ^**#**^p < 0.05 compared with the c-TR.

The agent U46619 produced concentration-dependent contraction in PVAT− and PVAT+ aortic rings with intact endothelium, and no alteration was verified in *E*_MAX_ (Figures 2I–L) and pEC_50_ values (Table 3) in any group.

When we compared only the PVAT− rings, the *E*_MAX_ was decreased in c-TR and o-TR when compared with c-SD and o-SD, respectively. These alterations were also observed in PVAT+ rings with no alterations in EC_50_ (Table 3).

**Figure 3:**
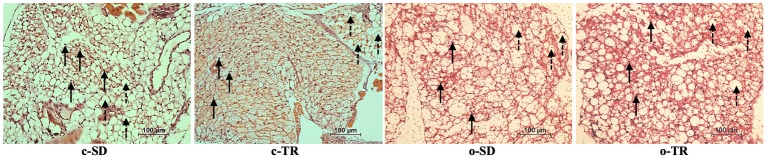
Representative histology of PVAT from mice thoracic aorta segments from sedentary (c-SD, *n* = 4), trained (c-TR, *n* = 6), obese sedentary (o-SD, *n* = 5), and obese trained (o-TR, *n* = 4) groups. Solid arrows indicate round nuclei and disrupted arrows indicate multilocular adipocytes. Adipocytes with larger lipid droplets in the o-SD group. Digital images were captured using the 40X objective. Scale bar = 100 μm.

The histology images demonstrated that PVAT surrounding thoracic aortas was composed of white adipose tissue (WAT) and brown adipose tissue (BAT), with nuclei and multilocular adipocytes. The qualitative analysis of hematoxylin and eosin stain showed visual difference in architecture and structure of PVAT in c-TR group, apparent predominance of BAT, when compared with c-SD group (Figure 3). On the other hand, the lipid droplets in o-SD group exhibited enlargement and coalescence alteration, apparent predominance of WAT, when compared with c-SD group (Figure 3). In o-TR group, the PVAT presented visual mix of WAT and BAT, with moderate architecture and structure alterations.

**Figure 4:**
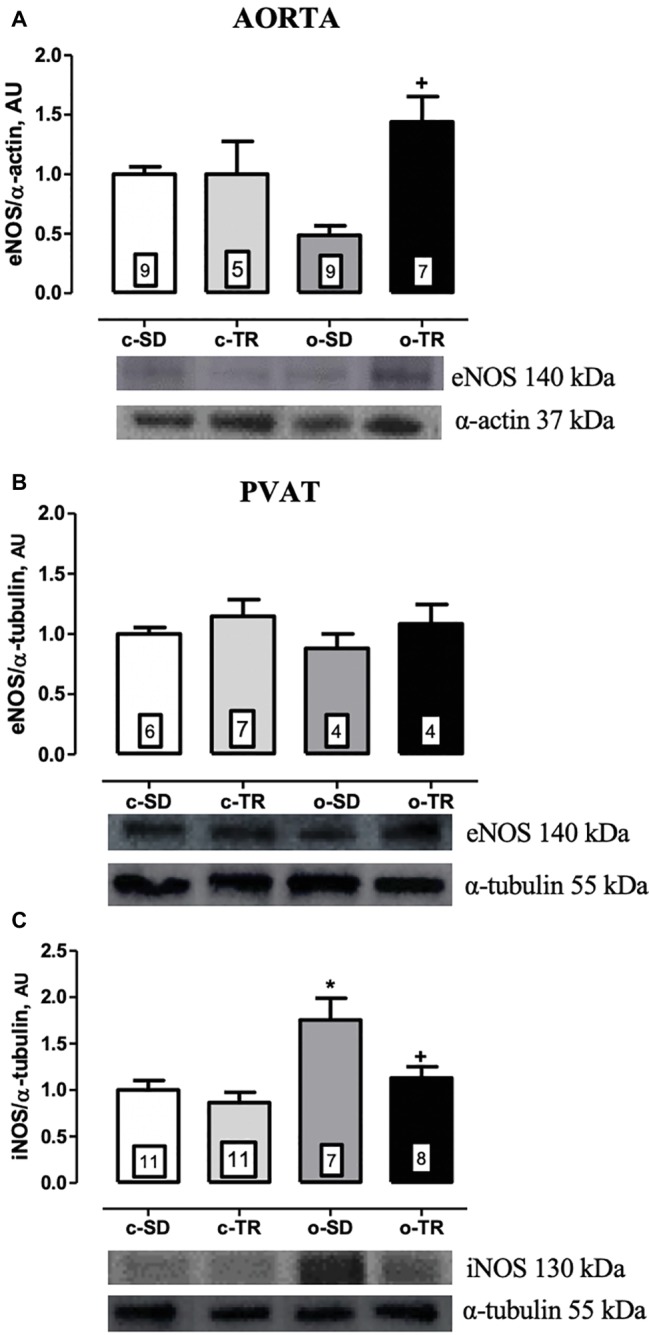
Protein expressions of endothelial nitric oxide synthase (eNOS: **A,B**) in thoracic aorta and PVAT of mice, respectively. Inducible nitric oxide synthase (iNOS: **C**) in PVAT of mice. Groups: sedentary (c-SD), trained (c-TR), obese sedentary (o-SD), and obese trained (o-TR). Bottom panel representative Western Blotting and top panel quantitative analysis. Data are presented as mean ± SEM. The number of animals per group is indicated in the figure. Two-way ANOVA: ^*^p < 0.05 compared with the c-SD; ^+^*p* < 0.05 compared with the o-SD.

The eNOS protein expression was significantly increased in thoracic aorta of the o-TR group (210%) compared with the o-SD group (Figure 4A). In contrast, in PVAT, we did not detect any alteration in protein expressions of eNOS (Figure 4B). The iNOS protein expression was increased in PVAT of the o-SD group (75%) compared with the c-SD group. Exercise training completely restored the expression of this protein in the o-TR group (Figure 4C). No change was found in c-TR group compared with c-SD group (Figure 4).

**Figure 5:**
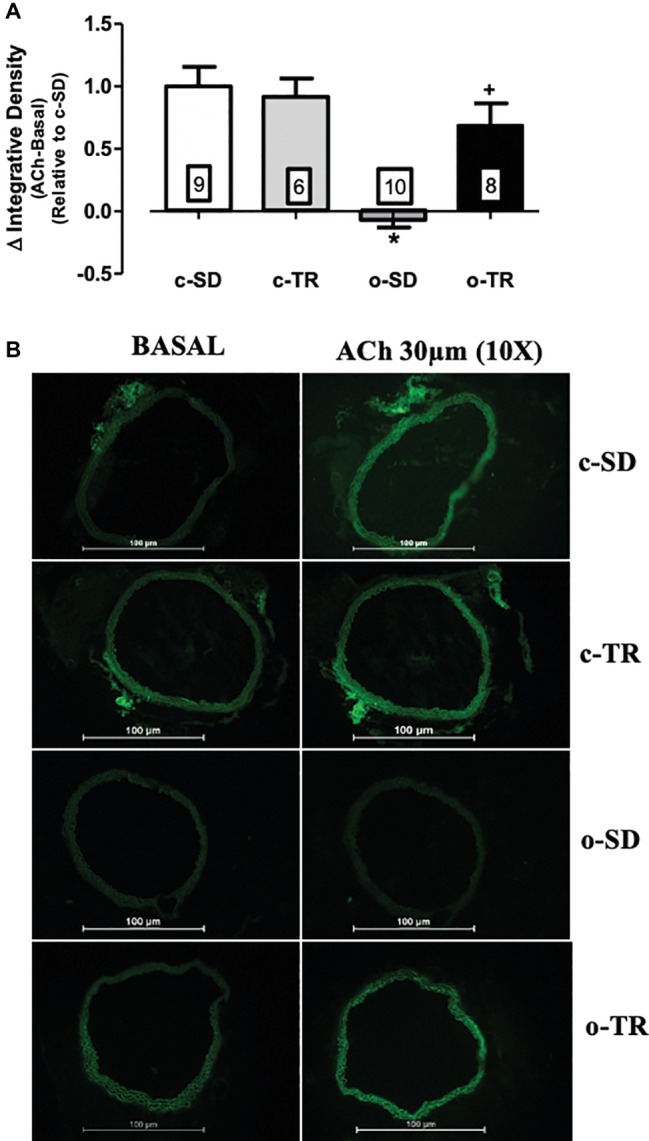
Nitric oxide (NO) production in thoracic aorta. Upper panel **(A)**: Quantitative analysis of the NO production measured by DAF-2 (delta of ACh integrative density minus basal integrative density) relative do c-SD in transverse sections of thoracic aorta. Lower panel **(B)**: Representative fluorographs of DAF-2-treated sections without (BASAL) or with acetylcholine (ACh, 30 µM) -stimulation of thoracic aorta from sedentary (c-SD), trained (c-TR), obese sedentary (o-SD), and obese trained (o-TR) mice. Data are presented as mean ± SEM. The number of animals per group is indicated in the figure. Two-way ANOVA: ^*^p < 0.05 compared with the c-SD; ^+^*p* < 0.05 compared with the o-SD.

The NO production induced by ACh (30 μM) was completely abolish in the o-SD group compared with the c-SD group, whereas aerobic exercise fully reestablished the NO production in the o-TR group (Figure 5A). The baseline NO production was not modified in all groups. No change was found in c-TR group when compared with c-SD group (Figure 5). We did not detect the fluorescence in PVAT using the methods described.

**Figure 6:**
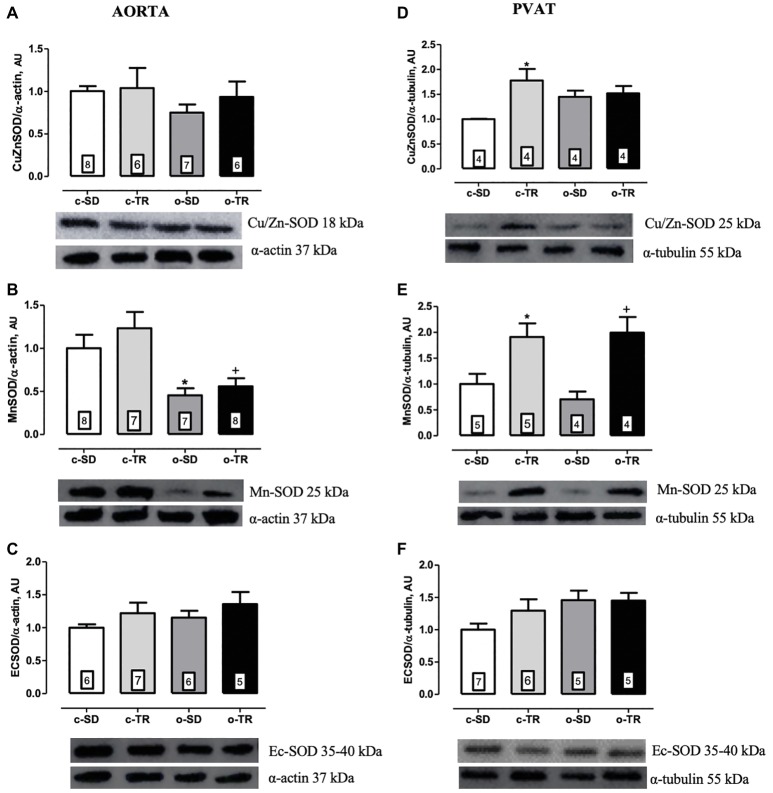
Protein expressions of cytosolic Cu/Zn-superoxide dismutase (Cu/ZnSOD: **A,D**), mitochondrial Mn-superoxide dismutase (Mn-OD: **B,E**) and extracellular superoxide dismutase (ECSOD: **C,F**) in thoracic aorta and PVAT of mice. Groups: sedentary (c-SD), trained (c-TR), obese sedentary (o-SD), and obese trained (o-TR). Bottom panel representative Western Blotting and top panel quantitative analysis. Data are presented as mean ± SEM. The number of animals per group is indicated in the figure. Two-way ANOVA: ^*^p < 0.05 compared with the c-SD; ^+^*p* < 0.05 compared with the o-SD.

**Figure 7:**
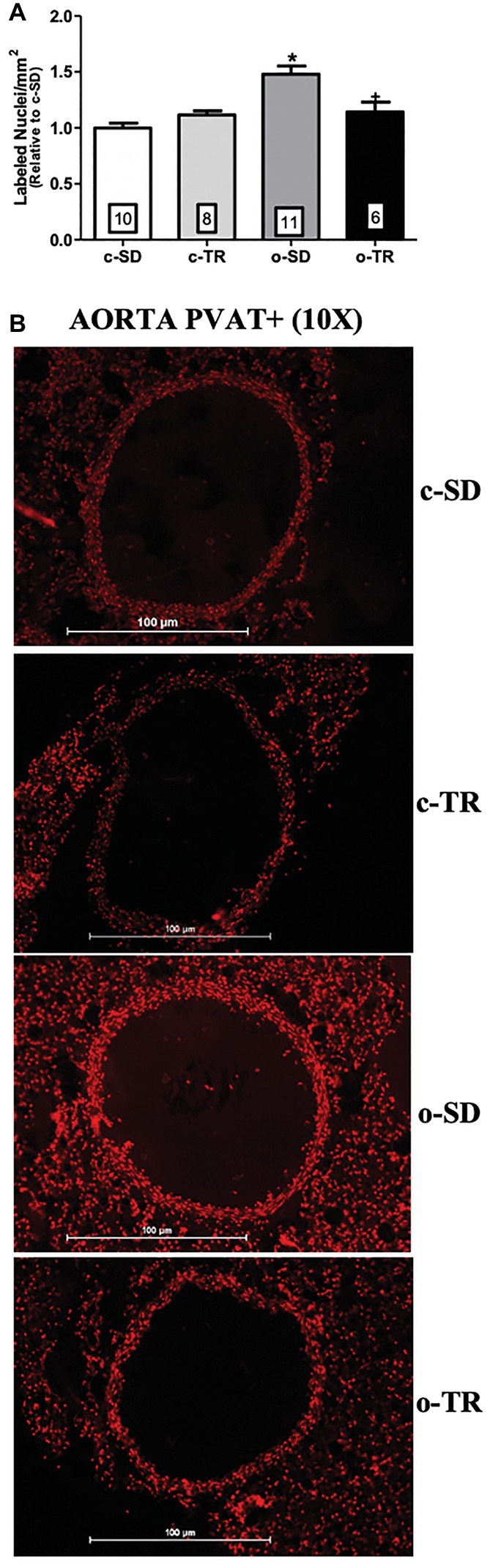
Reactive oxygen species (ROS) generation in thoracic aorta PVAT+. Quantitative analysis (upper panel: **A**) and representative fluorographs (lower panel: **B**) of the ethidium-bromide-positive nuclei in transverse sections of thoracic aorta PVAT+ (panel A), from sedentary (c-SD), trained (c-TR), obese sedentary (o-SD), and obese trained (o-TR) groups. Data are presented as mean ± SEM. The number of animals per group is indicated in the figure. Two-way ANOVA: ^*^p < 0.05 compared with the c-SD; ^+^*p* < 0.05 compared with the o-SD.

Finally, in thoracic aorta, we did not detect any alteration in protein expressions of Cu/Zn-SOD and Ec-SOD (Figures 6A,C, respectively). On the other hand, the aorta Mn-SOD protein expression was decreased in o-SD and o-TR groups (50 and 45%, respectively) compared with the c-SD and c-TR groups, respectively (Figure 6B). In PVAT tissue, the Cu/Zn-SOD protein expression was increased only in c-TR group when compared with c-SD group (Figure 6D). The Mn-SOD protein expression was increased in both trained groups, c-TR and o-TR, when compared with the c-SD and o-SD groups (Figure 6E). The protein expression of Ec-SOD was not modified in PVAT (Figure 6F). In addition, an increase in ROS generation in aorta and in the PVAT in the o-SD group (50%) compared with the c-SD group was observed. The elevated ROS generation was restored in o-TR group compared with the o-SD group (Figure 7A). No change was found in c-TR group compared with c-SD group (Figure 7).

## Discussion

Obesity is a complex disease strongly linked to the development of cardiovascular disease (CVD). It increases morbidity and mortality with a significative impact on the heath care systems. Endothelial dysfunction plays a crucial role in the pathophysiological process of microvascular and macrovascular complications of the metabolic disease (Koliaki et al., 2018). In the present study, our data show a significant increase in epididymal fat, total cholesterol, blood glucose, and insulin, demonstrating a metabolic alteration in the experimental model handled (o-SD group). Those changes were accompanied by an imbalance of pro-inflammatory circulating biomarkers, evidenced by elevated levels of leptin, resistin, and TNF-α. Furthermore, a significant increase in iNOS and decrease in Mn-SOD protein expressions in PVAT of thoracic aorta was observed and followed by a reduction in vascular NO production as well as a marked increase in vascular and PVAT ROS generation. Regarding morphological parameters, we also found visual enlargement and coalescence alteration of lipid droplets, associated with increased PVAT amount. Altogether, our study shows enhanced impairment of endothelium-dependent relaxation to ACh in the presence of PVAT, which was positively correlated with pro-inflammatory and redox state markers, without alterations in the relaxation response to SNP and contraction response to U46619.

Over the past decades, our concepts about adipose tissue (AT) have changed significantly and its biological function exhibits extreme complexity (Trayhurn, 2013). Adipose tissue macrophages (ATMs) are a major source of pro-inflammatory cytokines in obesity, which in turn recruit additional macrophages and propagates the chronic inflammation (Olefsky and Glass, 2010). The development of adipose tissue inflammation also starts with lipid accumulation and adipocyte hypertrophy, which increases adipocyte size (Bluher, 2016), leading to hypoxia caused by inadequate vascular support (Trayhurn, 2013). The hypoxia has been shown to increase even more the rate of infiltration of macrophages and the secretion of cytokines including TNF-α (O’Rourke et al., 2011), in accordance, in this study, we observed increased body weight and epididymal fat pad associated with elevated circulating TNF-α levels in the obese group (o-SD).

Evidence has demonstrated that aerobic exercise training is associated with a decrease in the risk of cardiovascular diseases and cardiovascular mortality (Nystoriak and Bhatnagar, 2018). In the presence of obesity, regular exercise contributes to alleviate the adverse consequences of the disease, including body weight and fat loss and metabolic and anti-oxidant improvement (Bigornia et al., 2010; Larson-Meyer et al., 2010). Additional studies show that regular exercise may preclude metabolic complications associated with obesity in the absence of weight loss (Samaan et al., 2014; Hespe et al., 2016; Petridou et al., 2018). Furthermore, it has been suggested that the functionality and integrity of AT are more important aspects for cardiometabolic risk than its total amount (Bastien et al., 2014; Koliaki et al., 2018). Our results show that aerobic exercise training for 8 weeks (o-TR group) was effective to ameliorate insulin levels, even though the reduction in hyperglycemia was not observed. Additionally, the circulatory level of TNF-α was decreased and a visual modification of morphological parameters was observed, without any change in body weight and fat mass.

The PVAT surrounds large arteries and veins, skeletal muscle micro vessels, and resistance vessels; however, cerebral vasculature is free of PVAT (Gil-Ortega et al., 2015). Furthermore, in micro vessels, the PVAT is an integral part of the vascular wall, not separated from the adventitial layer and may directly influence vascular function (Gil-Ortega et al., 2015). Morphological differences exist among PVAT depots; in murine thoracic aorta, the PVAT is BAT-like (Fitzgibbons et al., 2011). In obesity experimental models, the PVAT mass and adipocyte size are increased accompanied by other structural and functional modifications in PVAT (Marchesi et al., 2009; Araujo et al., 2018). The PVAT expands proportionally to the visceral fat pad and can trigger specific responses, leading to vascular dysfunction and cardiovascular disease (Fitzgibbons and Czech, 2014; Xia and Li, 2017). In the present study, we observed increased amount of PVAT and morphological alterations in lipid droplets associated with a dysfunction in endothelium-dependent relaxation in the thoracic aorta of obese mice. The inflammation and oxidative stress in PVAT promote a dysregulation of biomolecules production and vascular dysfunction (Omar et al., 2014; Xia et al., 2016). We evidenced an upregulation of iNOS protein expression in PVAT, and its activity and expression are related to inflammatory conditions (Codoner-Franch et al., 2011), as we demonstrated, simultaneously increased of ROS production could limit NO production and/or bioavailability. The macrophage infiltration in PVAT reduces adiponectin secretion (Bailey-Downs et al., 2013) and could be associated with endothelial dysfunction (Zhang et al., 2010). Our study failed to demonstrate any alteration in adiponectin concentration and additional studies are necessary once the controversial results remain in literature and have difficulties in associating circulatory adiponectin levels as a biomarker of cardiovascular disease and vascular dysfunction (Sattar et al., 2006; Sook Lee et al., 2013; Woodward et al., 2016).

It has been reported that PVAT releases substances responsible for affecting vascular tone, and it seems that exercise contributes to this process (Boa et al., 2017). Previous studies demonstrated that exercise training reduces PVAT inflammation (Lee et al., 2016; Ruffino et al., 2016). Aerobic exercise training stimulates angiogenesis in AT, improving in blood flow and reducing hypoxia and macrophage infiltration (You et al., 2013). It also prevents or attenuates infiltration of immune cells into PVAT improving vascular function (Boa et al., 2017). In parallel, the mechanical stimulus promoted by shear stress, in response to exercise, plays a fundamental role in the prevention of endothelial dysfunction (Laughlin and Roseguini, 2008) through reduction of ROS and increase in NO bioavailability (Lesniewski et al., 2011; Jendzjowsky and DeLorey, 2013). In the PVAT, the impact of exercise training induces the expression of eNOS demonstrating a key trigger of vascular recuperation in obese mice and reduction of ROS (Meziat et al., 2019). Our findings clearly showed that exercise training increased eNOS protein expression in aorta, as well as, prevented iNOS upregulation in PVAT. Regarding the redox state, exercise training increased Mn-SOD protein expression in PVAT, reducing tissue ROS generation. Finally, the relaxation response to ACh in the presence of PVAT and vascular NO production were fully reestablished by aerobic exercise.

## Conclusion

The presence of PVAT exacerbates endothelial dysfunction in thoracic aorta of obese mice associated with circulatory inflammation, upregulation of iNOS and oxidative stress in PVAT, and reduction of vascular NO production. Aerobic exercise training ameliorated the circulatory TNF-α levels, increased the anti-oxidant protein expressions, and decreased PVAT oxidative stress with beneficial impact on vascular relaxation.

## Data Availability

The raw data supporting the conclusions of this manuscript will be made available by the authors, without undue reservation, to any qualified researcher.

## Ethics Statement

The study protocol was approved (CEUA 3278-1) by the Ethics Committee for Animal Use of the University of Campinas (CEUA-UNICAMP, Brazil) and carried out in accordance with the ethical principles for animal experimentation adopted by the Brazilian Society of Laboratory Animal Science (SBCAL/COBEA).

## Author Contributions

ASS designed and executed the experiments, analyzed the data, wrote the manuscript. ACSS and CT executed the experiments, analyzed the data, and read the manuscript. MD conceived the study, guided the experimental design, data analysis and interpretation, and read and revised the manuscript.

### Conflict of Interest Statement

The authors declare that the research was conducted in the absence of any commercial or financial relationships that could be construed as a potential conflict of interest.
